# Association of Racial Bias With Burnout Among Resident Physicians

**DOI:** 10.1001/jamanetworkopen.2019.7457

**Published:** 2019-07-26

**Authors:** Liselotte Dyrbye, Jeph Herrin, Colin P. West, Natalie M. Wittlin, John F. Dovidio, Rachel Hardeman, Sara Emily Burke, Sean Phelan, Ivuoma Ngozi Onyeador, Brooke Cunningham, Michelle van Ryn

**Affiliations:** 1 Division of Community Internal Medicine, Department of Medicine, Mayo Clinic, Rochester, Minnesota; 2 Department of Internal Medicine, Yale School of Medicine, Charlottesville, Virginia; 3 Division of General Internal Medicine, Department of Medicine, Mayo Clinic, Rochester, Minnesota; 4 Department of Psychology, Yale University, New Haven, Connecticut; 5 School of Public Health, Division of Health Policy and Management, University of Minnesota, Minneapolis; 6 Department of Psychology, Syracuse University, Syracuse, New York; 7 Division of Health Care Policy and Research, Mayo Clinic, Rochester, Minnesota; 8 Department of Family Medicine and Community Health, University of Minnesota, Minneapolis; 9 School of Nursing, Oregon Health and Science University, Portland

## Abstract

**Importance:**

Burnout, a syndrome characterized by emotional exhaustion, depersonalization, and a decreased sense of efficacy, is common among resident physicians, and negative emotional states may increase the expression of prejudices, which are associated with racial disparities in health care. Whether racial bias varies by symptoms of burnout among resident physicians is unknown.

**Objective:**

To assess the association between burnout and explicit and implicit racial biases toward black people in resident physicians.

**Design, Setting, and Participants:**

This cohort study obtained data from surveys completed by first-year medical students and resident physicians in the United States as part of the Cognitive Habits and Growth Evaluation Study. Participants were followed up from enrollment in 2010 to 2011 through 2017. Participants completed questionnaires at year 4 of medical school as well as at the second and third years of residency. Only data from resident physicians who self-identified as belonging to a racial group other than black (n = 3392) were included in the analyses because of scarce evidence of racial bias in the care provided to black patients by black physicians. Resident physicians training in radiology or pathology were excluded because they provided less direct patient interaction.

**Main Outcomes and Measures:**

Burnout symptoms were measured by 2 single-item measures from the Maslach Burnout Inventory. Explicit attitudes about white and black people were measured by a feeling thermometer (FT, from 0 to 100 points, ranging from very cold or unfavorable [lowest score] to very warm or favorable [highest score]; included in the second-year [R2] and third-year [R3] questionnaires). The R2 Questionnaire included a racial Implicit Association Test (IAT; range: –2 to 2).

**Results:**

Among the 3392 nonblack resident physician respondents, 1693 (49.9%) were male, 1964 (57.9%) were younger than 30 years, and 2362 (69.6%) self-identified as belonging to the white race. In this cohort, 1529 of 3380 resident physicians (45.2%) had symptoms of burnout and 1394 of 3377 resident physicians (41.3%) had depression. From this group, 12 did not complete the burnout items and 15 did not complete the Patient-Reported Outcomes Measurement Information System (PROMIS) items. The mean (SD) FT score toward black people was 77.9 (21.0) and toward white people was 81.1 (20.1), and the mean (SD) racial IAT score was 0.4 (0.4). Burnout at the R2 Questionnaire time point was associated with greater explicit and implicit racial biases. In multivariable analyses adjusting for demographics, specialty, depression, and FT scores toward white people, resident physicians with burnout had greater explicit racial bias (difference in FT score, –2.40; 95% CI, –3.42 to –1.37; *P* < .001) and implicit racial bias (difference in IAT score, 0.05; 95% CI, 0.02-0.08; *P* = .002). A dose-response association was found between change in depersonalization from R2 to R3 Questionnaire and R3 Questionnaire explicit bias (for each 1-point increase the difference in R3 FT score decreased, –0.73; 95% CI, –1.23 to –0.23; *P* = .004) and change in explicit bias.

**Conclusions and Relevance:**

Among resident physicians, symptoms of burnout appeared to be associated with greater explicit and implicit racial biases; given the high prevalence of burnout and the negative implications of bias for medical care, symptoms of burnout may be factors in racial disparities in health care.

## Key Points

**Question:**

Are symptoms of burnout associated with resident physicians’ implicit and explicit biases toward black people?

**Findings:**

In this cohort study of 3392 second-year resident physicians who self-identified as nonblack, symptoms of burnout were associated with greater explicit and implicit racial biases. Recovery from burnout in the third year of residency was associated with the greatest reduction in explicit bias toward black people.

**Meaning:**

Given the high prevalence of burnout among resident physicians and the negative association between bias and suboptimal medical care, symptoms of burnout may be factors in disparities in care; the implications for the quality of care provided to black people and other disadvantaged groups could be substantial.

## Teaser

This study evaluates survey responses from a large national cohort of US resident physicians to assess the association between burnout and explicit and implicit racial biases among nonblack resident physicians.

## Introduction

Despite efforts on multiple fronts, substantial morbidity and mortality differences persist between white and black patients, regardless of their socioeconomic status and level of education.^1,2,3,4,5^ Although multiple complex factors are associated with this racial disparity in health status, the difference in medical care provided by physicians to black patients compared with white patients is a substantial aspect.^6,7,8,9,10,11^ Previous studies have found that, although physicians consciously value equitable care,^12,13^ their directly expressed (explicit) and unconscious (implicit) biases are factors in their behaviors and decisions that are associated with the medical care they actually provide.^14,15,16,17,18,19,20,21,22^ These data, coupled with evidence of racial bias among trainees,^23,24,25^ have led to calls for graduate medical education to include curricula focused on understanding and addressing racial health disparities.^26,27,28^

Burnout is prevalent among resident physicians^29,30,31^ and is an underrecognized threat to the success of curricular interventions. A substantial body of literature has documented a high prevalence of burnout and depression among resident physicians.^32,33^ Burnout, a syndrome characterized by emotional exhaustion, depersonalization (ie, cynicism), and a decreased sense of efficacy, is job related, situation specific, and largely driven by work-related factors.^34^ Among resident physicians, the primary drivers of burnout include work intensity, suboptimal supervisor behaviors, lack of flexibility and control, educational debt, and work-home conflict.^33^ Physicians’ negative emotional states have been shown to be associated with greater explicit racial bias in medical decision-making.^35^ Negative emotions, like those characterized by burnout and depression, also can impede cognitive performance,^36,37,38^ making implicit biases more likely to play a role in behaviors and decision-making.^39^ Burnout, as a negative emotional state, could activate bias, reduce cognitive capacity leading to inappropriate application of heuristics, or have negative consequences in mindful decision making in other ways.

To our knowledge, the potential association between burnout and explicit and implicit racial biases in resident physicians has not been previously studied. Therefore, we undertook a longitudinal study to assess this association in a national sample of resident physicians, who had been followed up since their first year of medical school as participants in the Cognitive Habits and Growth Evaluation Study (CHANGES).

## Methods

The institutional review boards of the University of Minnesota, Oregon Health and Sciences University, and Mayo Clinic approved this study. Written informed consent was provided by all CHANGES participants. Methods and results are reported in accordance with the Strengthening the Reporting of Observational Studies in Epidemiology (STROBE) guidelines for cohort and cross-sectional studies^40^ and with the American Association for Public Opinion Research (AAPOR) guidelines for surveys.^41^

**Figure 1.**
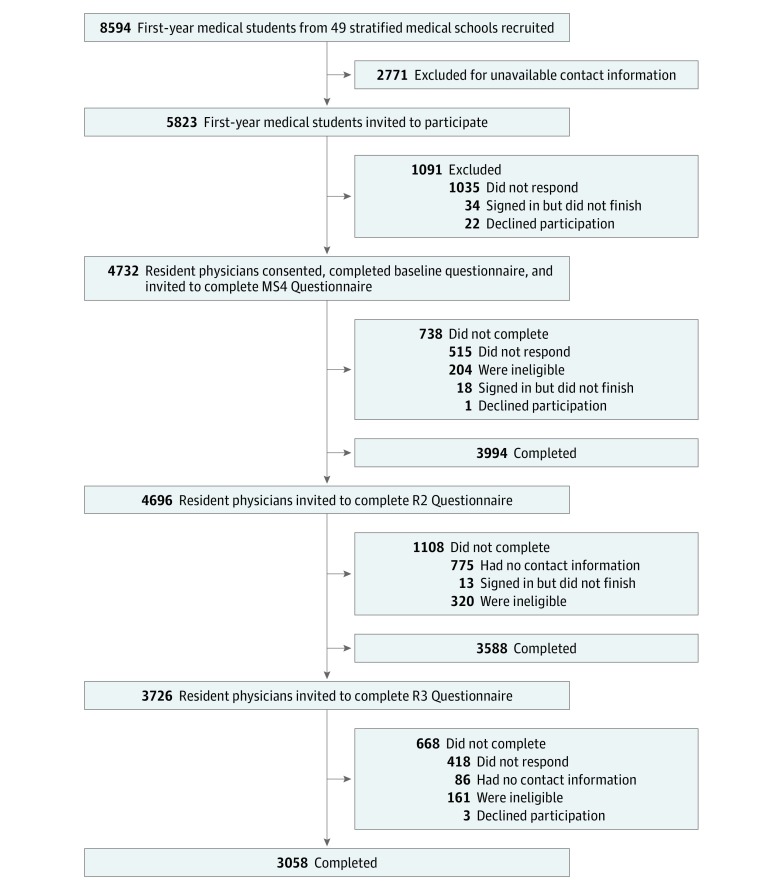
CHANGES Participant Recruitment Flowchart This recruitment flow represents the entire Cognitive Habits and Growth Evaluation Study (CHANGES) cohort, which includes black residents. Of the cohort, 3588 completed the survey; 196 self-identified as black and were not included in this analysis, resulting in 3392 nonblack residents. From this group, 12 did not complete the burnout items (n = 3380) and 15 did not complete the Patient-Reported Outcome Measurement Information System (PROMIS) items (n = 3377). MS4 indicates medical school year 4; R2, second year of residency; and R3, third year of residency.

The methods used in CHANGES have been previously reported.^29,42,43,44,45,46,47,48^ Briefly, in 2010 to 2011, first-year medical students attending a stratified random sample of 49 allopathic US medical schools were invited to participate in CHANGES (Figure 1). In 2014, medical students who had consented and provided baseline data (ie, baseline respondents) were invited to complete the year 4 of medical school questionnaire (MS4 Questionnaire). Subsequently, in 2016, baseline respondents were invited to complete the second year of residency questionnaire (R2 Questionnaire). In 2017, resident physicians who had completed the MS4 Questionnaire and were not training in radiology or pathology (specialties excluded because they provided less direct patient interaction) were invited to complete the third year of residency questionnaire (R3 Questionnaire).

At each time point, written informed consent was provided by the respondent. The last year of follow-up was 2017. Participants received financial compensation for each questionnaire they completed.

We included data from only nonblack resident physicians (ie, individuals who defined themselves as belonging to a racial group other than black). Black resident physicians were excluded because there was less evidence of racial bias in the care provided to black patients by black physicians.^20,49,50^

### Measures

The questionnaires (eAppendix in the Supplement) included questions about demographic characteristics (year of birth, sex, race, ethnicity, relationship status, and parental status), specialty, burnout, depression, and attitudes about black and white people.

#### Burnout and Depressive Symptoms

Consistent with approaches used in other large studies,^29,30,51,52^ we measured symptoms of burnout on the R2 Questionnaire and the R3 Questionnaire using 2 single-item measures (a question on emotional exhaustion, and another on depersonalization) adapted from the full 22-item Maslach Burnout Inventory and with 7 levels of responses ranging from never to every day. Previous studies including several independent samples of more than 10 000 physicians and medical students have demonstrated that these 2 single-item measures stratify the risk of burnout.^53,54^ In these studies, the likelihood ratios for the once-a-week or the more-often response to the emotional exhaustion measure ranged from 6 to 42 and to the depersonalization measure ranged from 16 to 37 compared with the respective scale of the full Maslach Burnout Inventory.^53,54^ In addition, the positive predictive value of high levels of emotional exhaustion was 88.2% and depersonalization was 89.6%; the area under the receiver operating characteristic curve for the emotional exhaustion measure was 0.94 and for the depersonalization measure was 0.93, in comparison to the full Maslach Burnout Inventory domain scores.^53,54^

In these previous studies, dichotomized overall burnout (indicated by a high score, defined as having weekly or more often symptoms of either emotional exhaustion or depersonalization) was associated with patient care and physician well-being outcomes, with magnitudes of association similar to those of overall burnout as measured by the full Maslach Burnout Inventory.^53,54^ In this present study, we used overall burnout based on the single items in our primary analyses. We repeated the same analyses using the emotional exhaustion and depersonalization scales as continuous variables (the findings are reported in eTable 1 in the Supplement). We measured symptoms of depression on the R2 Questionnaire using the depression short form 4a of PROMIS (Patient-Reported Outcomes Measurement Information System), an instrument developed and validated by the National Institutes of Health.^55^ Respondents were asked to rate how often in the past 7 days they experienced affective and cognitive manifestations of depression. Response options were never, almost never, sometimes, fairly often, and very often with scores ranging from 4 to 20. The area under the receiver operating characteristic curve for detecting individuals with major depression, as diagnosed by the 9-item Patient Health Questionnaire, was 0.90.^56^ Participants were considered to have depressive symptoms if they had PROMIS depression scores of 8 or higher (sensitivity of 83.1% and specificity of 84.3%).^56^

#### Explicit and Implicit Racial Biases

On the R2 and R3 Questionnaires, participants indicated their feelings about black (African American in the survey instrument) individuals on a feeling thermometer (FT), by moving a slider along a scale from 0 to 100 points, ranging from very cold or unfavorable (lowest score) to very warm or favorable (highest score). Respondents answered similar questions regarding their feelings about white (Caucasian in the survey instrument) people. An FT has been established as valid for measuring attitudes about various social groups^57^ and has been used to assess attitudes toward black people.^18,58^ Explicit bias about black people was captured by adjusting FT scores toward black people for FT scores toward white people.

The R2 Questionnaire also included an Implicit Association Test (IAT; range: –2 to 2) to measure implicit bias toward black people compared with implicit bias toward white people (a positive score indicates greater prowhite bias).^59^ During the IAT, participants sorted pictures of people of European and African origin and words (eg, *beautiful*, *cheerful*, *friend*, *failure*, *tragic*, and *scorn*). In one block, participants were instructed to categorize images and words either as *white people* or *good* or as *black people* or *bad*; in the other block, they were instructed to categorize images and words either as *white people* or *bad* or as *black people* or *good*. Relative preference for white women and white men over black women and black men (IAT D score) was calculated by subtracting the mean response latency for the former IAT practice trials from the mean response latency for the latter practice trials and then dividing by the SD for all practice trials.^60^ The IAT score ranges from –2 (strong preference for black men and women) to 2 (strong preference for white women and men). Previous studies have used the race IAT to measure implicit racial bias.^19,20,61^ The IAT was not included in the R3 Questionnaire.

### Statistical Analysis

Data analysis was conducted from March 1, 2018, through December 21, 2018, and then again from April 30, 2019, to May 7, 2019. Response rates at each time point were calculated using standard methods.^41^ In addition to evaluating basic summary statistics of respondent characteristics, FT scores, and IAT scores, we assessed the differences in FT scores between the R2 and R3 Questionnaires using paired *t* tests. Using analysis of variance models, we assessed the bivariate association between high emotional exhaustion, high depersonalization, overall burnout (high emotional exhaustion and/or high depersonalization), and symptoms of depression as well as racial implicit and explicit biases at the R2 Questionnaire time point; we reported the mean (SD) bias score for each response group and the overall *P* value for each categorical variable. For dichotomous variables, we also estimated the mean (95% CI) of the difference in bias scores between the groups. Then, to assess the independent association of burnout and depression with implicit and explicit biases at the R2 Questionnaire time point, we estimated for each bias measure a multivariable regression model that included indicators for burnout and depression as well as age, sex, race, ethnicity, relationship status, parental status, and specialty; we reported coefficients with 95% CIs for each burnout and depression group and overall *P* values for each variable. The explicit bias in the second year of residency model also included the R2 Questionnaire FT score toward white people as a covariate.

To examine the association of changes in symptoms of burnout with changes in explicit bias, we classified R2 and R3 Questionnaire respondents into chronic burnout (symptoms of burnout at both the second and third years of residency time points), never burned out (did not have symptoms of burnout at either time point), recovered from burnout (had symptoms of burnout at the second but not third year of residency time point), and new burnout (had symptoms of burnout only at the third year of residency time point). We then estimated a regression model in which R3 Questionnaire FT score toward black people was the dependent variable and burnout pattern was the independent variable, adjusting for age, sex, race, ethnicity, relationship status, parental status, specialty, R2 Questionnaire reported depression, R2 Questionnaire FT score toward white people, and R2 Questionnaire FT score toward black people.

In secondary analyses, we replicated the main analyses using the components of burnout (emotional exhaustion and depersonalization) as separate indicator variables and, alternatively, as continuous scales. We also included depression score as a continuous variable. For the long-term analyses, these variables were entered as changes in emotional exhaustion, depersonalization, and depression scores.

The original CHANGES cohort was selected using a stratified sampling design in which all medical students at geographically diverse set of schools were invited to participate; however, we have not incorporated sampling or nonresponse rates into the current analysis. Not doing so implies that estimates and inferences are valid only for the survey respondents and not for the general population of resident physicians; however, we selected this approach because respondents are now grouped by residency program rather than by school, with medical school explaining almost none of the variance in any of the key dependent or independent variables in the present study. Two-sided *P* < .05 were interpreted as statistically significant. All analyses were performed with Stata, version 15.1 (StataCorp LLC).

## Results

### Cross-sectional Cohort

The demographic characteristics and specialty training distribution of the 3392 nonblack second-year resident physicians (Table 1) were generally similar to those of all resident physicians in the United States.^62,63^ Of the 3392 participants, 1693 (49.9%) were male, 1964 (57.9%) were younger than 30 years, and 2362 (69.6%) self-identified as belonging to the white race.

**Table 1.** Demographic Characteristics of Cohort of 3392 Nonblack Second-Year Resident Physicians

Variable	No. (%)
Sex	
Male	1693 (49.9)
Female	1683 (49.6)
Other	7 (0.2)
Missing data	9 (0.3)
Age	
<30 y	1964 (57.9)
≥30 y	1401 (41.3)
Missing data	27 (0.8)
Race	
East Asian	446 (13.1)
South Asian	320 (9.4)
White	2362 (69.6)
Multiracial	140 (4.1)
Other^a^	124 (3.7)
Missing data	0
Ethnicity	
Hispanic/Latino	3202 (94.4)
Not Hispanic/Latino	168 (5.0)
Missing data	22 (0.6)
Have children	
Yes	447 (13.2)
No	2904 (85.6)
Missing data	41 (1.2)
Relationship status	
Single	1881 (55.5)
Married/with partner	1375 (40.5)
Separated/widowed	59 (1.7)
Missing data	77 (2.3)
Second-year residency specialty^b^	
Surgery	822 (24.2)
Primary care	1456 (42.9)
Other direct	870 (25.6)
Nondirect	236 (7.0)
Other	8 (0.2)

^a^ Other race included American Indian/Alaska Native, Native Hawaiian/ Pacific Islander, or indicated unknown.

^b^ Specialty included surgery (eg, general, subspecialty, otolaryngology, neurosurgery, and obstetrics); primary care (eg, family medicine, internal medicine, and pediatrics); other direct (eg, dermatology, emergency medicine, neurology, physical medicine, preventive medicine, and psychiatry); nondirect (eg, radiology, nuclear medicine, and pathology); and other.

In this cross-sectional cohort of 3380 resident physicians, 1203 (35.6%) had high emotional exhaustion, with a mean (SD) score of 3.0 (1.6) on the single item for emotional exhaustion; 1179 (34.9%) had high depersonalization, with a mean (SD) score of 2.9 (1.7) on the single item for depersonalization; and 1529 (45.2%) had burnout. Depressive symptoms were present in 1394 of 3377 resident physicians (41.3%), and the mean (SD) score on the PROMIS scale was 7.1 (3.3). Mean (SD) FT score toward black people was 77.9 (21.0), mean (SD) FT score toward white people was 81.1 (20.1), and mean (SD) racial IAT score was 0.4 (0.4), all of which indicate a preference for white people over black people. Histograms of R2 Questionnaire FT scores toward black people and white people, R2 Questionnaire IAT scores, and a scatterplot of FT score toward black people compared with FT score toward white people can be found in eFigure 1, eFigure 2, eFigure 3, and eFigure 4, respectively, in the Supplement.

**Figure 2.**
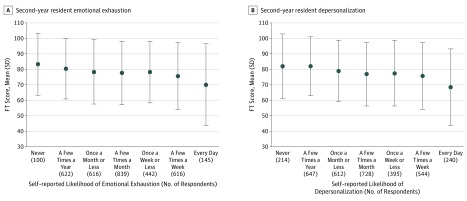
Feeling Thermometer (FT) Score Toward Black People Significant differences in FT score across levels of distress are seen. The FT score is obtained by moving a slider along a scale from 0 to 100 points, ranging from very cold or unfavorable (lowest score) to very warm or favorable (highest score).

Higher emotional exhaustion and depersonalization scores were associated with more unfavorable attitudes toward black people (as indicated by lower FT scores) (Figure 2). Resident physicians with high emotional exhaustion had lower mean (SD) FT scores toward black people compared with resident physicians without high emotional exhaustion (75.9 [21.9] vs 78.9 [20.4]; difference, –3.0; 95% CI, –4.5 to –1.5; *P* < .001). Similarly, resident physicians with high depersonalization had lower mean (SD) FT scores toward black people compared with resident physicians without high depersonalization (74.8 [22.3] vs 79.5 [20.1]; difference, –4.7; 95% CI, –6.2 to –3.2; *P* < .001). Overall, resident physicians who had at least 1 symptom of burnout had lower mean (SD) FT scores toward black people compared with those without symptoms of burnout (75.9 [21.9] vs 79.5 [20.1]; difference, –3.6; 95% CI, –5.0 to –2.2; *P* < .001). Resident physicians with depressive symptoms also had lower mean (SD) FT scores toward black people (74.9 [22.2] vs 80.0 [19.8]; difference, –5.0; 95% CI, –6.5 to –3.6; *P* < .001). On multivariable analysis, burnout (difference in FT score, –2.40; 95% CI, –3.42 to –1.37; *P* < .001; Table 2) and, in particular, depersonalization (for each 1-point increase, the difference in FT score decreased, –0.83; 95% CI, –1.22 to –0.45; *P* < .001; eTable 1 in the Supplement) were independently associated with lower FT score toward black people.

**Table 2.** Multivariable Analysis to Identify Factors Associated With Explicit and Implicit Biases Among Second-Year Resident Physicians, Cross-sectional Cohort^a^

Variable	Explicit Bias^b^		Implicit Bias^c^	
	Coefficient (95% CI)	*P* Value	Coefficient (95% CI)	*P* Value
Symptoms of burnout^d^		<.001		.002
No	1 [Reference]		1 [Reference]	
Yes	−2.40 (−3.42 to −1.37)		0.05 (0.02 to 0.08)	
Symptoms of depression^e^		.17		.98
No	1 [Reference]		1 [Reference]	
Yes	−0.73 (−1.77 to 0.32)		−0.00 (−0.03 to 0.03)	
Age category		<.001		<.001
<30 y	1 [Reference]		1 [Reference]	
≥30 y	2.58 (1.55 to 3.60)		−0.06 (−0.09 to −0.03)	
Sex		.001		.07
Male	1 [Reference]		1 [Reference]	
Female	1.74 (0.74 to 2.75)		−0.04 (−0.07 to −0.01)	
Other	−7.43 (−18.80 to 3.95)		−0.05 (−0.39 to 0.28)	
Hispanic or Latino		.09		.22
No	1 [Reference]		1 [Reference]	
Yes	1.93 (−0.33 to 4.18)		−0.04 (−0.11 to 0.03)	
White race		.50		.53
No	1 [Reference]		1 [Reference]	
Yes	−0.38 (−1.48 to 0.72)		0.01 (−0.02 to 0.04)	
Married or partnered		.07		.20
No	1 [Reference]		1 [Reference]	
Yes	1.03 (−0.07 to 2.12)		0.02 (−0.01 to 0.05)	
Have children		.14		.13
Yes	1 [Reference]		1 [Reference]	
No	1.20 (−0.41 to 2.82)		−0.04 (−0.09 to 0.01)	
Specialty		.02		.20
Surgery	1 [Reference]		1 [Reference]	
Primary	0.57 (−0.69 to 1.82)		−0.03 (−0.07 to 0.01)	
Other direct	−0.73 (−2.12 to 0.67)		−0.04 (−0.08 to 0.00)	
Nondirect	−2.28 (−4.39 to −0.18)		−0.00 (−0.06 to 0.06)	
FT Score toward white people	0.76 (0.74 to 0.79)	<.001	NA	NA

Abbreviation: FT, feeling thermometer; NA, not applicable.

^a^ Data exclude black respondents.

^b^ As measured by FT score (range: 1-100). Lower score (more negative) indicates less favorable feelings toward black people and greater explicit bias. Scores are adjusted for FT scores toward white people.

^c^ As measured by racial implicit attitude test (IAT). Higher score (more positive) indicates greater implicit racial bias. The IAT score ranges from −2 (strong preference for black women and men) to +2 (strong preference for white women and men).

^d^ Positive for symptoms of burnout if a score of 5 (≥once per week) or higher (range, 1-7) on either of 2 questions taken from the Maslach Burnout Inventory. One question was on emotional exhaustion, and the other was on depersonalization.

^e^ Positive for symptoms of depression if a score of ≥8 on the PROMIS (Patient-Reported Outcome Measurement Information System) depression short form 4a.

Implicit bias toward black people was also greater among resident physicians with high depersonalization (racial IAT mean [SD] scores, 0.48 [0.41] vs 0.42 [0.42]; difference, 0.05; 95% CI, 0.02-0.09; *P* < .001) and overall burnout (mean [SD], 0.47 [0.42] vs 0.42 [0.42]; difference, 0.05; 95% CI, 0.02-0.07; *P* = .002). No statistically significant difference in implicit bias toward black people was found among resident physicians with or without high emotional exhaustion (mean [SD], 0.46 [0.42] vs 0.43 [0.42]; difference, 0.03; 95% CI, 0.00-0.06; *P* = .07) and with or without depressive symptoms (mean [SD], 0.44 [0.42] vs 0.44 [0.42]; difference, 0.00; 95% CI, –0.03 to 0.03; *P* = .82). On multivariable analysis, burnout (difference in IAT score, 0.05; 95% CI, 0.02-0.08; *P* = .002; Table 2) and, in particular, depersonalization (for each 1-point increase, the difference in IAT score increased, 0.02; 95% CI, 0.01-0.03; *P* < .001; eTable 1 in the Supplement) were independently associated with implicit bias toward black people.

### Long-term Cohort

In 2017, a total of 3058 resident physicians completed the R3 Questionnaire, among whom 2888 (94.4%) had completed the R2 Questionnaire. The 144 respondents who indicated their race was black were excluded, resulting in 2744 resident physicians comprising the long-term cohort. The demographic characteristics of the resident physicians providing long-term data were similar to those who provided only R2 Questionnaire data on age, race, ethnicity, parental status, and relationship status; however, women were more likely to provide long-term data (279 women respondents [43.1%] for R2 Questionnaire only vs 1404 women respondents [51.2%] for R2 and R3 Questionnaires; *P* = .001). The cohort had differences in specialty, as resident physicians who indicated on the R2 Questionnaire that they were training in pathology and radiology were not invited to complete the R3 Questionnaire (eTable 2 in the Supplement).

Among the 2733 resident physicians in this long-term cohort, 884 (33.3%) had symptoms of chronic burnout, 381 (13.9%) had recovered from symptoms of burnout, 346 (10.2%) had new symptoms of burnout, and 1122 (41.1%) never had symptoms of burnout. Mean (SD) FT scores toward black people increased from the R2 Questionnaire time point to the R3 Questionnaire time point (77.9 [21.0] vs 80.9 [20.0]; mean difference, 2.9 [19.2]; *P* < .001). Mean (SD) FT scores toward white people also increased from the R2 Questionnaire to the R3 Questionnaire time point (81.0 [20.1] vs 82.3 [19.4]; mean difference, 1.1 [19.4]; *P* < .01).

Mean (SD) FT scores toward black people at each time point for resident physicians who had symptoms of chronic burnout, had recovered from burnout, had new burnout, and never had burnout are shown in eFigure 5 in the Supplement. Resident physicians who never had burnout had higher mean FT scores toward black people at both R2 and R3 Questionnaire time points (80.2 and 82.7) compared with those who recovered from burnout (76.2 and 81.2), had new burnout (78.0 and 79.7), and had chronic burnout (76.0 and 78.9). Resident physicians who recovered from burnout had the highest gain in mean FT scores toward black people over the course of 1 year (4.8) compared with those who never had burnout (2.8), had new burnout (1.6), and had chronic burnout (2.9).

On multivariable analysis, the R3 Questionnaire FT score toward black people did not differ statistically across different burnout change patterns from the 2 time points, although the most favorable point-estimate implication was seen among resident physicians experiencing recovery from burnout (referent never had burnout; δ FT scores: recovered from burnout, 0.82 [95% CI, –1.17 to 2.80]; new burnout, –1.78 [95% CI, –3.83 to 0.28]; chronic burnout, –1.10 [95% CI, –2.69 to 0.48]; overall *P* = .10). However, a dose-response association was found between change in depersonalization from R2 to R3 Questionnaires and R3 Questionnaire explicit bias (for each 1-point increase in depersonalization, the difference in R3 FT score was –0.73; 95% CI, –1.23 to –0.23; *P* = .004) and change in explicit bias (Table 3).

**Table 3.** Multivariable Analysis to Identify Factors Associated With Explicit Bias Among Third-Year Resident Physicians, Long-term Cohort

Variable	R3 Explicit Bias^a^		Change in Explicit Bias^a^	
	Coefficient (95% CI)	*P* Value	Coefficient (95% CI)	*P* Value
EE delta^b^	−0.54 (−1.10 to 0.01)	.053	−0.63 (−1.22 to −0.04)	.04
DP delta^c^	−0.73 (−1.23 to −0.23)	.004	−0.76 (−1.29 to −0.22)	<.01
EE delta × DP delta	−0.09 (−0.33 to 0.16)	.50	−0.07 (−0.34 to 0.19)	.56
PROMIS delta^d^	0.04 (−0.18 to 0.26)	.71	−0.01 (−0.24 to 0.23)	.94
Age		.17		.01
<30 y	1 [Reference]		1 [Reference]	
≥30 y	−0.94 (−2.27 to 0.40)		−1.83 (−3.25 to −0.41)	
Sex		.32		.14
Male	1 [Reference]		1 [Reference]	
Female	−0.56 (−1.85 to 0.72)		−1.27 (−2.64 to 0.10)	
Other	−9.37 (−23.81 to 5.07)		−7.17 (−22.57 to 8.24	
Hispanic or Latino		.15		.25
No	1 [Reference]		1 [Reference]	
Yes	2.18 (−0.77 to 5.13)		1.86 (−1.28 to 5.01)	
White race		<.001		<.001
No	1 [Reference]		1 [Reference]	
Yes	2.76 (1.33 to 4.20)		2.99 (1.46 to 4.52)	
Married or partnered		.63		.32
No	1 [Reference]		1 [Reference]	
Yes	−0.35 (−1.76 to 1.07)		−0.76 (−2.27 to 0.75)	
Have children		.45		.46
Yes	1 [Reference]		1 [Reference]	
No	0.83 (−1.28 to 2.94)		0.85 (−1.40 to 3.10)	
Specialty at R2		<.01		.04
Surgery	1 [Reference]		1 [Reference]	
Primary	0.31 (−1.26 to 1.87)		−0.01 (−1.68 to 1.66)	
Other direct	−2.34 (−4.09 to −0.59)		−2.02 (−3.88 to −0.15)	
Nondirect	2.79 (−4.37 to 9.94)		4.04 (−3.60 to 11.67)	
FT Score toward white people at R2	−0.05 (−0.10 to −0.00)	.046	−0.39 (−0.42 to −0.35)	<.001
FT Score toward black people at R2	0.56 (0.52,0.61)	<.001	NA	NA

Abbrevations: DP, depersonalization; EE, emotional exhaustion; FT, feeling thermometer; NA, not applicable; PROMIS, Patient-Reported Outcome Measurement Information System; R2, second year of residency; R3, third year of residency.

^a^ As measured by FT score (range: 1-100). Lower score (more negative) indicates less favorable feelings toward black people and greater explicit bias. Scores are adjusted for FT scores toward white people. Change in explicit bias score is difference between second year and third year of residency FT score.

^b^ Change in response to the 1 question on emotional exhaustion from the Maslach Burnout Inventory between R2 and R3 Questionnaire time points. Range is 1 to 7, with higher scores indicating greater emotional exhaustion.

^c^ Change in response to the 1 question on depersonalization from the Maslach Burnout Inventory. Range is 1 to 7, with higher score indicative of greater depersonalization.

^d^ Change in response to the PROMIS depression short form 4a between R2 and R3 Questionnaire time points. Scores range from 4 to 20, with higher scores indicating worse symptoms.

## Discussion

In this large national study of US resident physicians, reported symptoms of burnout were associated with greater explicit and implicit biases toward black people. Generally, feelings toward black people became more favorable from the second to the third year of residency. Worsening of depersonalization symptoms was statistically significantly associated with explicit bias toward black people.

These findings suggest that resident physicians’ feelings toward black people can become more favorable over the course of 1 year. This improvement may be associated with positive experiences with black people (colleagues, coworkers, and patients), graduate medical education efforts to reduce racial biases, changing signals of contextual antibias norms, and other factors.^47,58,64^ Whether the association between burnout and bias is causal is unclear, and both burnout and bias may be associated with the factors just described. However, these findings are consistent with those of other studies, which reported that positive emotions are associated with decreases in bias,^65,66^ suggesting that successful efforts to reduce symptoms of burnout among resident physicians may be useful in reducing health care inequalities.

We also found that implicit racial bias was higher among resident physicians with symptoms of burnout. According to previous studies, physicians with higher implicit bias toward black people demonstrate fewer patient-centered behaviors during clinical interactions with black patients; in turn, their black patients have greater distrust, have lower level of adherence to treatment recommendations, and are less likely to follow up.^6,13,18,67,68,69,70,71,72,73,74,75,76^ These data suggest symptoms of burnout may be associated with negative outcomes for black patients.

If the association between burnout and bias toward black people is present among physicians in practice or after residency, it may be a factor in the explicit use of race in medical decision-making. As the prevalence of burnout symptoms among practicing physicians exceeds 40%,^77^ the implications for the quality of care provided to black people, as well as to other disadvantaged groups, could be substantial. Further study is warranted to establish whether the association between burnout and racial bias persists among physicians in practice.

The findings of this study add to previous studies showing burnout as a threat to safe, high-quality care.^78^ Unfortunately, because little is known about the most effective strategies to reduce the prevalence of burnout,^79,80^ additional research into mitigating work-related drivers of burnout is needed.

### Limitations

This study has several limitations. First, we assessed only a limited number of factors that may be associated with explicit and implicit biases. Second, we relied on measures of bias rather than on observed behaviors. Third, although the findings suggest an association between symptoms of burnout and bias toward black people, the magnitude of the observed associations was small to medium,^81^ and we could not ascertain if these associations were causal. In addition, minimal clinically important differences for FT and IAT scores have not been established in this population. Fourth, the generalizability of the results is unknown. However, the participation and questionnaire response rates were high, and resident physicians attended a wide range of medical schools and training programs. The demographic characteristics and specialty training distribution of the cohort were generally similar to all US medical residents. Fifth, differences in sex and specialty distributions were observed between respondents to only R2 Questionnaire and respondents to both R2 and R3 Questionnaires (eTable 2 in the Supplement). These differences may be explained by the men being less likely than the women in this study to complete questionnaires and the R3 Questionnaire not being sent to resident physicians who were training in radiology or pathology (excluded because they had less direct patient interaction).

## Conclusions

Among US resident physicians, explicit bias and implicit bias were associated with symptoms of burnout. Given the high prevalence of burnout among physicians and the negative implications of bias for medical care, symptoms of burnout may be factors in disparities in care. The implications for the quality of care provided to black people and other disadvantaged groups could be substantial.

## Notes

Supplement. **eAppendix.** Cognitive Habits and Growth Evaluation Study (CHANGES) Questionnaire Items Relevant to “Association of Racial Bias with Burnout Among Resident Physicians.” **eFigure 1.** Histogram of Feeling Thermometer Score Toward Black People from the Second Year of Residency Questionnaire **eFigure 2.** Histogram of Feeling Thermometer Score Toward White People from the Second Year of Residency Questionnaire **eFigure 3.** Histogram of Race Implicit Attitude Test from the Second Year of Residency Questionnaire **eFigure 4.** Scatterplot of Feeling Thermometer Score Toward Black People Versus Feeling Thermometer Score Toward White People, Second Year of Residency Questionnaire **eFigure 5.** Mean Feeling Thermometer Score Toward Black People at Second Year and Third Year of Residency for Residents with Symptoms of Chronic Burnout, Recovered from Burnout, New Burnout, and Never Had Burnout **eTable 1.** Multivariable Analysis to Identify Factors Associated with Explicit and Implicit Bias Against Black People Among Second-year Residents, Cross-sectional Cohort **eTable 2.** Demographic Characteristics of the Residents Providing Longitudinal Data (Second- and Third-year Survey Responders) and Residents Providing Only Cross-sectional Data (Second-year Responders Only)
