# Supplementary material for: Association of Racial Bias With Burnout Among Resident Physicians
Source: JAMA Netw Open. 2019 Jul 26;2(7):e197457. doi: 10.1001/jamanetworkopen.2019.7457 (PMC6661712; doi:10.1001/jamanetworkopen.2019.7457)
Supplement: Supplement. — eAppendix. Cognitive Habits and Growth Evaluation Study (CHANGES) Questionnaire Items Relevant to “Association of Racial Bias with Burnout Among Resident Physicians.” eFigure 1. Histogram of Feeling Thermometer Score Toward Black People from the Second Year of Residency Questionnaire eFigure 2. Histogram of Feeling Thermometer Score Toward White People from the Second Year of Residency Questionnaire eFigure 3. Histogram of Race Implicit Attitude Test from the Second Year of Residency Questionnaire eFigure 4. Scatterplot of Feeling Thermometer Score Toward Black People Versus Feeling Thermometer Score Toward White People, Second Year of Residency Questionnaire eFigure 5. Mean Feeling Thermometer Score Toward Black People at Second Year and Third Year of Residency for Residents with Symptoms of Chronic Burnout, Recovered from Burnout, New Burnout, and Never Had Burnout eTable 1. Multivariable Analysis to Identify Factors Associated with Explicit and Implicit Bias Against Black People Among Second-year Residents, Cross-sectional Cohort eTable 2. Demographic Characteristics of the Residents Providing Longitudinal Data (Second- and Third-year Survey Responders) and Residents Providing Only Cross-sectional Data (Second-year Responders Only) [file jamanetwopen-2-e197457-s001.pdf]

Supplementary Online Content

Dyrbye L, Herrin J, West CP, et al. Association of racial bias with burnout among resident physicians. *JAMA Netw Open*. 2019;2(7): e197457. doi:10.1001/jamanetworkopen.2019.7457

eAppendix. Cognitive Habits and Growth Evaluation Study (CHANGES) Questionnaire Items Relevant to "Association of Racial Bias With Burnout Among Resident Physicians."

eFigure 1. Histogram of Feeling Thermometer Score Toward Black People From the Second Year of Residency Questionnaire

eFigure 2. Histogram of Feeling Thermometer Score Toward White People From the Second Year of Residency Questionnaire

eFigure 3. Histogram of Race Implicit Attitude Test From the Second Year of Residency Questionnaire

eFigure 4. Scatterplot of Feeling Thermometer Score Toward Black People Versus Feeling Thermometer Score Toward White People, Second Year of Residency Questionnaire

eFigure 5. Mean Feeling Thermometer Score Toward Black People at Second Year and Third Year of Residency for Residents With Symptoms of Chronic Burnout, Recovered From Burnout, New Burnout, and Never Had Burnout

eTable 1. Multivariable Analysis to Identify Factors Associated With Explicit and Implicit Bias Against Black People Among Second-year Residents, Cross-sectional Cohort

eTable 2. Demographic Characteristics of the Residents Providing Longitudinal Data (Second- and Third-year Survey Responders) and Residents Providing Only Cross-sectional Data (Second-year Responders Only)

This supplementary material has been provided by the authors to give readers additional information about their work.

eAppendix. Cognitive Habits and Growth Evaluation Study (CHANGES) Questionnaire Items Relevant to “Association of Racial Bias with Burnout Among Resident Physicians.”

Demographics

Age - Calculated from: What is your year of birth?

Please indicate your gender.

- Male (1)
- Female (2)
- Other - please describe (3)

Ethnicity - Please indicate your ethnicity.

- Hispanic or Latino (1)
- Not Hispanic or Latino (2)
- Unknown (3)

Race - Please indicate your race. Choose *ALL* that apply.

- American Indian/Alaska Native
- East Asian
- South Asian
- Black
- Native Hawaiian/ Pacific Islander
- White
- Other (please describe)

A ‘Race’ categorical variable was computed from the individual items. If more than one race was checked participants were put in the multi-racial category.

Children

- Yes (1)
- No (2)

Relationship Status Choose *ALL* the apply.

- Never married
- Single
- Have a boyfriend
- Have a girlfriend
- Separated
- Divorced
- Widowed
- Engaged
- Married or domestic partner
- Living with romantic partner
- Have more than one romantic partner

What residency or fellowship program are you in now? (Please select from the list below).

- Anesthesiology
- Allergy/Immunology
- Child Neurology (Neurology)
- Dermatology
- Diagnostic Radiology/Nuclear Medicine
- Emergency Medicine
- Emergency Medicine/Family Medicine
- Family Medicine
- Family Medicine/Preventive Medicine
- Internal Medicine
- Internal Medicine/Anesthesiology
- Internal Medicine/Dermatology

Internal Medicine/Emergency Medicine
Internal Medicine/Family Practice
Internal Medicine/Medical Genetics
Internal Medicine/Neurology
Internal Medicine/Pediatrics
Internal Medicine/Preventive Medicine
Internal Medicine/Psychiatry
Neurodevelopmental Disabilities (Neurology)
Neurological Surgery
Neurology
Nuclear Medicine
Obstetrics and Gynecology
Orthopaedic Surgery
Otolaryngology
Pathology-Anatomic and Clinical
Pediatrics
Pediatrics/Anesthesiology
Pediatrics/Dermatology
Pediatrics/Emergency Medicine
Pediatrics/Medical Genetics
Pediatrics/Physical Medicine and Rehabilitation
Pediatrics/Psychiatry/Child and Adolescent Psychiatry
Physical Medicine and Rehabilitation
Plastic Surgery
Plastic Surgery-Integrated
Preventive Medicine
Psychiatry
Psychiatry/Family Practice
Psychiatry/Neurology
Radiation Oncology
Radiology-Diagnostic
Surgery-General
Thoracic Surgery-Integrated
Transitional Year
Urology
Vascular Surgery-Integrate
Ophthalmology
Other

Measures:

Burnout

Two item burnout measure

PROMIS Depression

[Scale – 1 Never; 2 Almost Never; 3 Sometimes; 4 Fairly often; 5 Very Often]

In the last 7 days - I felt worthless.
In the last 7 days - I felt helpless.
In the last 7 days - I felt depressed.
In the last 7 days - I felt hopeless.

The CHANGES study used the HealthMeasures Scoring Service (https://www.assessmentcenter.net/ac_scoringservice)

Selections: Custom Short Form, Measurement System [PROMIS], Select Respondents [Adults], Select Domain [Emotional distress – Depression/Sadness], PROMIS Bank v1.0 – Depression, Select Calibration Sample [PROMIS Wave 1 – Default]

Feeling Thermometers:

(Range: 0 – very cold to 100 – very warm)
Below you will see categories of people with sliders next to them. Indicate how you feel towards each group by moving the slider all the way to the left (very cold or unfavorable), all the way to the right (very warm or favorable), or somewhere in between.

	**Very cold or unfavorable**	**Very warm or favorable**
African Americans		
Caucasians		

©2019 Dyrbye L et al. *JAMA Network Open*

eFigure 1. Histogram of Feeling Thermometer Score Toward Black People from the Second Year of Residency Questionnaire

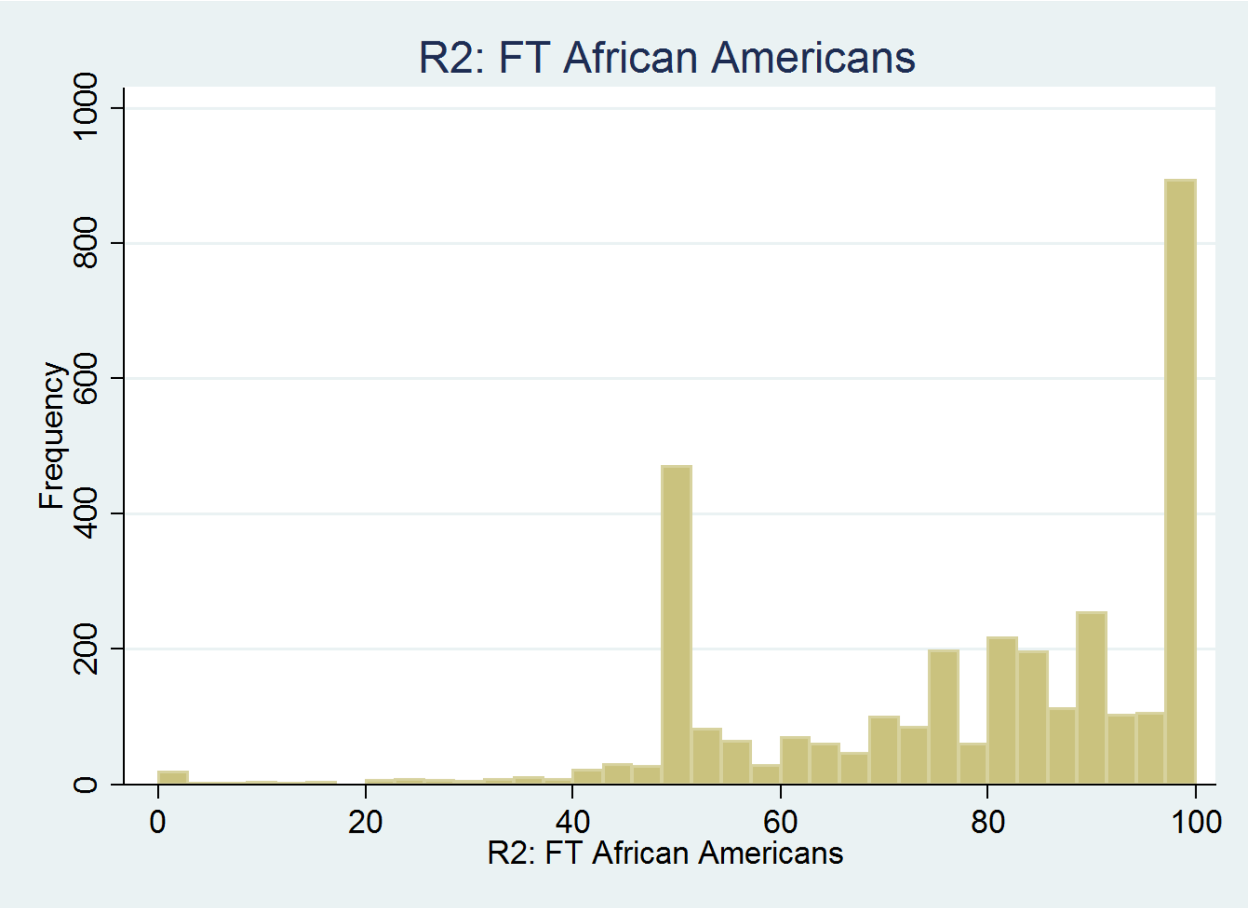

Abbreviations: R2, second year of residency; FT, feeling thermometer score

©2019 Dyrbye L et al. *JAMA Network Open*

eFigure 2. Histogram of Feeling Thermometer Score Toward White People from the Second Year of Residency Questionnaire

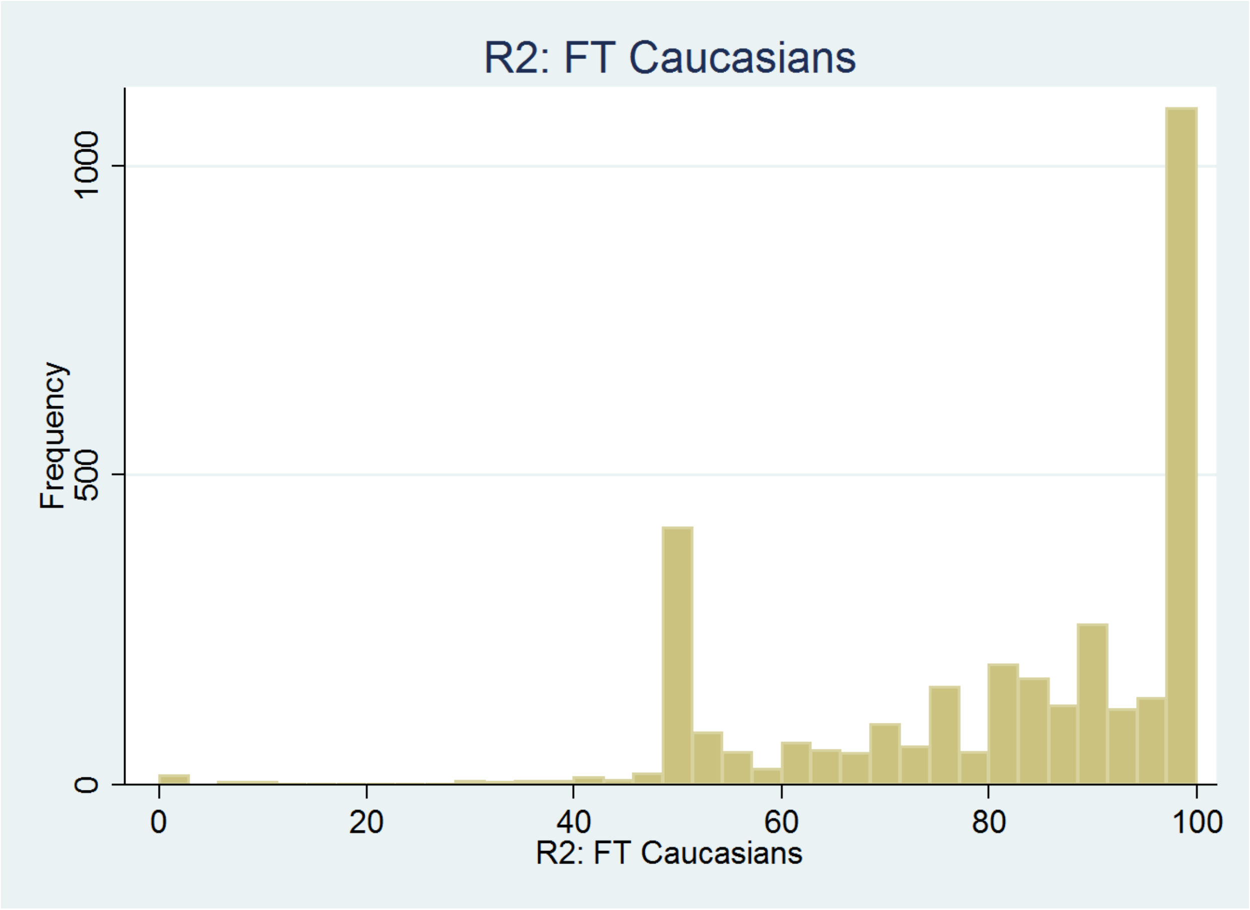

Abbreviations: R2, second year of residency; FT, feeling thermometer score

©2019 Dyrbye L et al. *JAMA Network Open*

eFigure 3. Histogram of Race Implicit Attitude Test from the Second Year of Residency Questionnaire

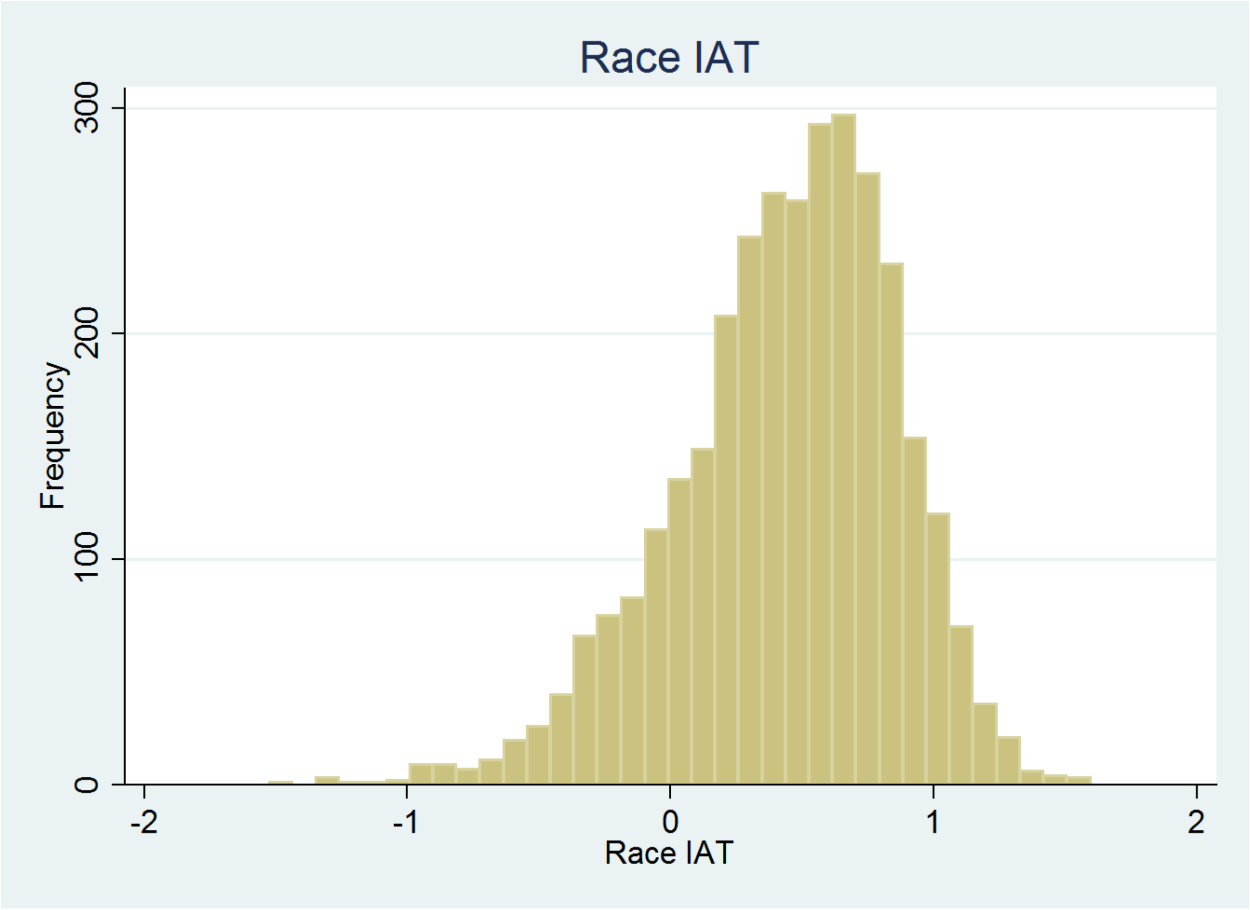

Abbreviations: IAT, Implicit Attitude Test

©2019 Dyrbye L et al. *JAMA Network Open*

eFigure 4. Scatterplot of Feeling Thermometer Score Toward Black People Versus Feeling Thermometer Score Toward White People, Second Year of Residency Questionnaire

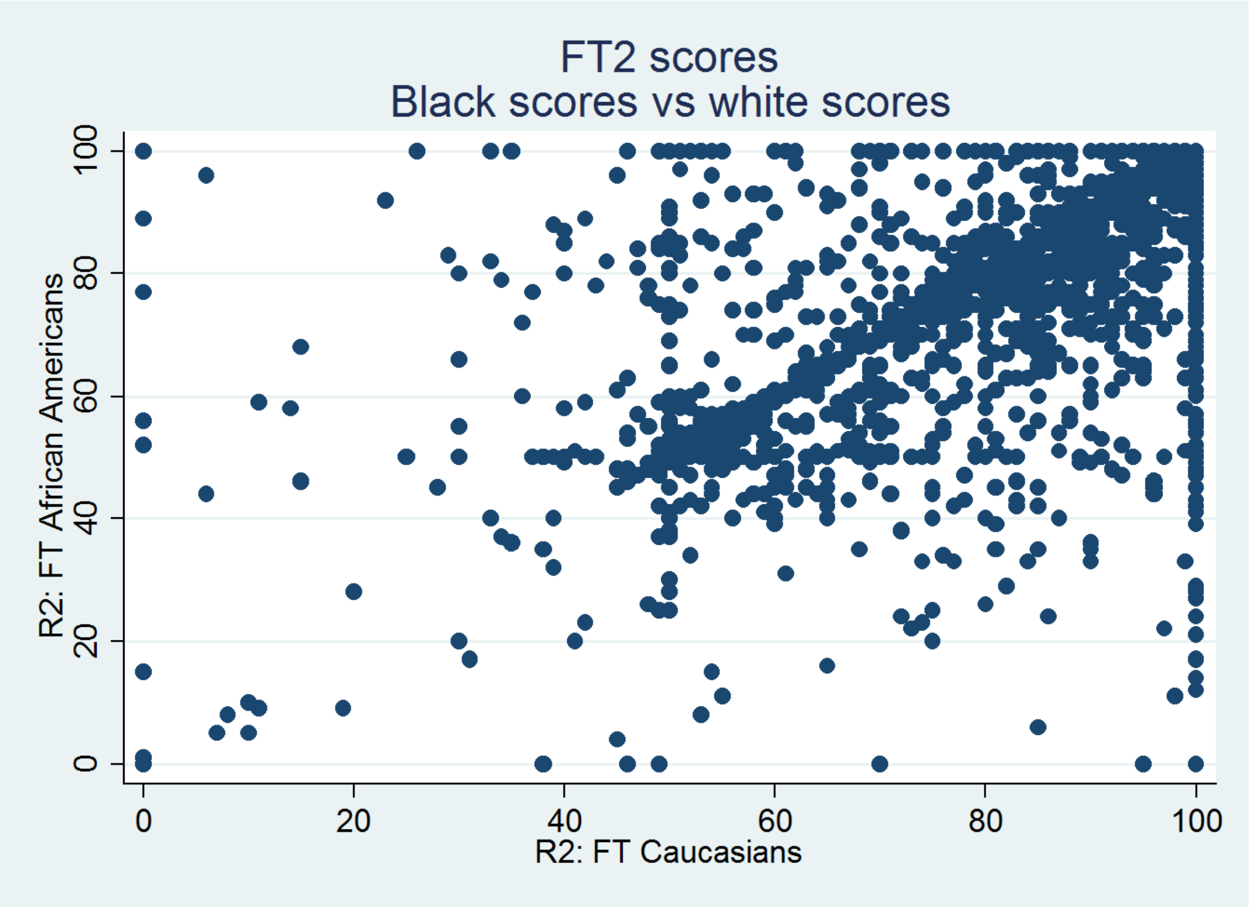

Abbreviations: R2, second year of residency; FT2, second year of residency feeling thermometer score; FT, feeling thermometer score

©2019 Dyrbye L et al. *JAMA Network Open*

eFigure 5. Mean Feeling Thermometer Score Toward Black People at Second Year and Third Year of Residency for Residents with Symptoms of Chronic Burnout, Recovered from Burnout, New Burnout, and Never Had Burnout

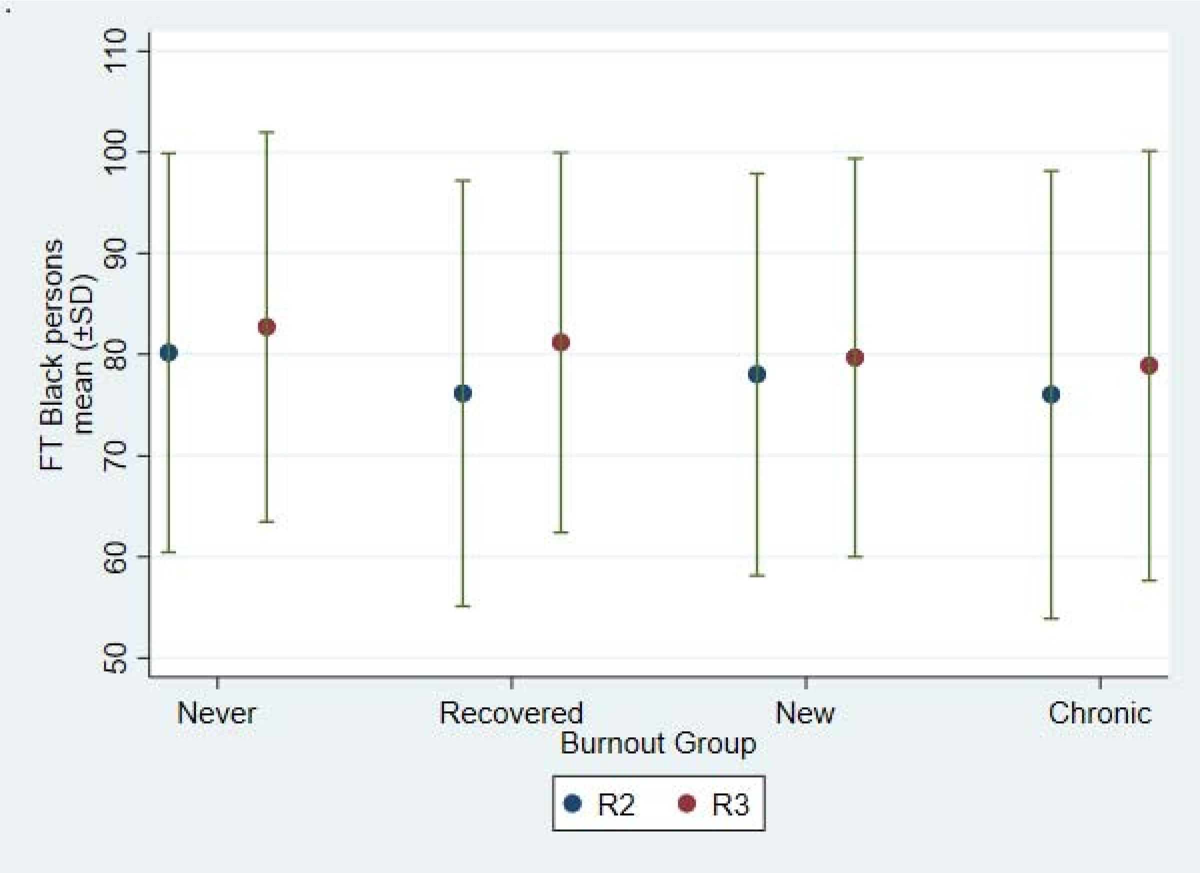

Abbreviations: R2, second year of residency; R3, third year of residency; FT, feeling thermometer score

©2019 Dyrbye L et al. *JAMA Network Open*

eTable 1. Multivariable Analysis to Identify Factors Associated with Explicit and Implicit Bias Against Black People Among Second-year Residents, Cross-sectional Cohort

Variable	Explicit Bias [1]		Implicit Bias [2]	
	Coeff (95% CI)	P-value	Coeff (95% CI)	P-value
Emotional Exhaustion score[3]	-0.08 (-0.52,0.37)	0.74	-0.00 (-0.01,0.01)	0.89
Depersonalization score[4]	-0.83 (-1.22,-0.45)	<0.001	0.02 (0.01,0.03)	<0.001
Depression score[5]	-0.14 (-0.31,0.03)	0.11	-0.00 (-0.01,0.00)	0.33
Age category		<0.001		<0.001
<30	ref		ref	
30+	2.59 (1.57,3.62)		-0.06 (-0.09,-0.03)	
Gender		0.002		0.14
Male	ref		ref	
Female	1.66 (0.65,2.67)		-0.03 (-0.06,-0.00)	
Other	-7.23 (-18.58,4.11)		-0.05 (-0.39,0.28)	
Hispanic or Latino		0.07		0.19
No	ref		ref	
Yes	2.09 (-0.16,4.33)		-0.05 (-0.12,0.02)	
White race		0.54		0.55
No	ref		ref	
Yes	-0.35 (-1.45,0.75)		0.01 (-0.02,0.04)	
Married or Partnered		0.12		0.18
No	ref		ref	
Yes	0.88 (-0.22,1.97)		0.02 (-0.01,0.06)	
Have children		0.12		0.12
Yes	ref		ref	
No	1.29 (-0.32,2.90)		-0.04 (-0.09,0.01)	
Specialty		0.01		0.21
Surgery	ref		ref	
Primary	0.37 (-0.89,1.63)		-0.03 (-0.07,0.01)	
Other Direct	-0.95 (-2.34,0.45)		-0.04 (-0.08,0.01)	
Non Direct	-2.71 (-4.82,-0.60)		0.01 (-0.06,0.07)	
Feeling thermometer score toward white people	0.75 (0.73,0.78)	<0.001		

Data excludes Black respondents

[1] As measured by feeling thermometer score. Lower score (more negative score) indicates less favorable feelings towards black people and greater explict bias. Scores are adjusted for feeling thermometer scores towards white people. Range is 0-100 point scale.

[2] As measured by race implicit attitude test. Higher score (more positive score) indicates greater implicit racial bias. 58 The IAT score ranges from -2 (strong preference for Black men and women) to +2 (strong preference for White women and men).

[3] Measured using one question on “emotional exhaustion” from the Maslach Burnout Inventory. Range is 1 to 7, with higher score indicative of greater emotional exhaustion.

[4] Measured using one question on “depersonalization” from the Maslach Burnout Inventory. Range is 1 to 7, with higher score indicative of greater depersonalization.

©2019 Dyrbye L et al. *JAMA Network Open*

[5] Measured using the Patient-Reported Outcome Measurement Information System depression short form 4a. Scores range from 4 to 20 with higher scores representing worse symptoms.

©2019 Dyrbye L et al. *JAMA Network Open*

eTable 2. Demographic Characteristics of the Residents Providing Longitudinal Data (Second- and Third-year Survey Responders) and Residents Providing Only Cross-sectional Data (Second-year Responders Only)

**Characteristic**	**Second Year Responder only** (n = 648)	**Second and Third Year Responder** (n = 2744)	**P-value**
**Gender**			0.001
Male	364 (56.2)	1329 (48.4)	
Female	279 (43.1)	1404 (51.2)	
Other	2 (0.3)	5 (0.2)	
Missing	3 (0.5)	6 (0.2)	
**Age**			0.40
Less than 30	364 (56.2)	1600 (58.3)	
30 or older	276 (42.6)	1125 (41.0)	
Missing	8 (1.2)	19 (0.7)	
**Race**			0.15
American Indian or Alaska Native	0 (0.0)	1 (0.0)	
East Asian	81 (12.5)	365 (13.3)	
South Asian	70 (10.8)	250 (9.1)	
Native Hawaiian/ Pacific Islander	434 (67.0)	1928 (70.3)	
White	27 (4.2)	87 (3.2)	
Unknown[1]	32 (4.9)	108 (3.9)	
MultiRacial	16 (4.4)	108 (3.9)	
Missing[2]	0	0	
**Ethnicity**			0.71
Hispanic/Latino	611 (94.3)	2591 (94.4)	
Not Hispanic/Latino	34 (5.2)	134 (4.9)	
Missing	3 (0.5)	19 (0.7)	
**Had children at R2**			0.47
Yes	89 (13.7)	358 (13.0)	
No	537 (82.9)	2367 (86.3)	
Missing	22 (3.4)	19 (0.7)	
**Relationship Status at R2**			0.52
Single	359 (55.4)	1522 (55.5)	
Married/Partner	245 (37.8)	1130 (41.2)	
Separated/Widowed	13 (2.0)	46 (1.7)	
Missing	31 (4.8)	46 (1.7)	
**Specialty at R2[3]**			-
Surgery	108 (16.7)	714 (26.0)	
Primary	197 (30.4)	1259 (45.9)	
Other Direct	123 (19.0)	747 (27.2)	

©2019 Dyrbye L et al. *JAMA Network Open*

Characteristic	Second Year Responder only (n = 648)	Second and Third Year Responder (n = 2744)	P-value
Non Direct	214 (33.0)	22 (0.8)	
Other	6 (0.9)	2 (0.1)	

Abbreviations: R2, second year of residency questionnaire
[1] Respondent indicated unknown race.
[2] Response was considered missing if the respondent did not answer the question.
[3] Specialty: Surgery (e.g., general, subspecialty, otolaryngology, neurosurgery, obstetrics); Primary Care (e.g., family medicine, internal medicine, pediatrics); Other Direct (e.g., dermatology, emergency medicine, neurology, physical medicine, preventative medicine, psychiatry), Non-Direct (e.g., radiology, nuclear medicine, pathology), and Other. As residents who were training in radiology or pathology were excluded the chi-squared test on specialty is not relevant. The 22 residents in the Non Direct category who responded to the PGY-2 and PGY-3 surveys were largely training in internal medicine at the PGY-2 time-point. Between the PGY-2 and PGY-3 surveys they moved into another residency program that was classified as Non Direct. We chose to keep these individuals in the sample as they saw patients at the PGY-2 time-point.

©2019 Dyrbye L et al. *JAMA Network Open*
